# Epidemiology, Morbidity and Mortality Associated With Anesthesia in Early Life: A Subgroup Analysis of the German NEonate and Children audiT of Anesthesia pRactice IN Europe (NECTARINE) Cohort

**DOI:** 10.1002/pan.70115

**Published:** 2026-01-16

**Authors:** Claudia Neumann, Alina Schenk, Ehrenfried Schindler, Karin Becke‐Jakob, German Nectarine Group Collaborators

**Affiliations:** ^1^ Department of Anesthesiology and Intensive Care Medicine University Hospital Bonn Bonn Germany; ^2^ Institute of Medical Biometry, Informatics and Epidemiology University Hospital Bonn Bonn Germany; ^3^ Department of Anesthesiology University Children's Hospital Basel Basel Switzerland

**Keywords:** critical events, neonatal anesthesia, neonates, patient safety

## Abstract

**Background:**

The NEonate and Children audiT of Anesthesia pRactice IN Europe (NECTARINE) study, led by the ESAIC Clinical Trials Network, collected prospective data on 5609 children up to 60 weeks postmenstrual age undergoing 6542 anesthetic procedures across 165 centers in 31 European countries (ESAIC_CTN_NECTARINE). While the study provides comprehensive European data, healthcare systems, perioperative practices, and organizational standards vary considerably between countries. Germany was selected a priori for a dedicated subcohort analysis due to its substantial contribution (10.3% of the total dataset) and notable differences to other European countries in the absence of a centralization and national training program in pediatric anesthesia. This focused evaluation aimed to benchmark national data against European findings, identify country‐specific strengths and weaknesses, and support targeted quality improvement and guideline development within the German healthcare system.

**Aims:**

To compare the incidence, nature, and consequences of perioperative critical events between the German and non‐German NECTARINE cohorts and to assess practice‐related risk factors and outcomes.

**Methods:**

Data from 14 German centers were analyzed using mixed‐effects logistic regression to examine associations between critical events and 30‐day morbidity and mortality. Perioperative management practices and risk profiles were compared with those from the rest of the European cohort.

**Results:**

The German cohort showed a significantly higher rate of critical events (47.0% vs. 33.9%, *p* < 0.001), with cardiovascular instability being most frequent (82.6%). Within German centers, the occurrence of a critical event nearly tripled the risk of postoperative complications within 30 days (OR: 2.85; 95% CI: 1.67–4.87). ASA status and number of surgeries were also significant predictors of morbidity.

**Conclusions:**

This prospectively defined subanalysis demonstrates that perioperative outcomes and practice patterns in Germany differ from European averages, particularly regarding the frequency of critical events, thresholds for intervention, staffing ratios, and complication profiles. These insights highlight the need for targeted interventions in German pediatric anesthesia, contribute to contextualizing European data, and offer baseline data for future cross‐border quality initiatives and trials.

**Trial Registration:**

ClinicalTrails.gov NCT02350348

## 1 Introduction

### 1.1 Background/Rationale

The anesthesiologic care of prematurely born and term infants poses significant challenges to anesthesia teams due to their increased susceptibility to intra‐ and post‐operative critical events, which contribute to elevated morbidity and mortality rates [1, 2].

The increasing survival rate of prematures is an additional cause as the incidence of premature births has risen, partly driven by advancing parental age, leading to a growing number of surgical interventions required for this vulnerable population [3, 4]. Premature infants often present with conditions such as necrotizing enterocolitis or congenital anomalies, necessitating specialized anesthetic care. The APRICOT study highlighted that the anesthesiologist's experience plays a crucial role in improving perioperative outcomes in children from birth to 15 years of age [1]. Yet, interpreting physiological parameters and identifying triggers for complications in neonates remain complex and underexplored.

These areas have been explored in detail in only a few studies, often focusing on specific patient groups [5, 6, 7, 8]. To address these challenges, the European Society of Anesthesiology and Intensive Care (ESAIC) Clinical Trials Network initiated the NEonate and Children audiT of Anesthesia pRactice IN Europe (NECTARINE) study (ESAIC_CTN_NECTARINE).

This large, multicenter prospective observational study gathered standardized perioperative data on 5609 children up to 60 weeks postmenstrual age undergoing 6542 anesthetic procedures across 165 centers in 31 European countries. Given the variability in healthcare systems, perioperative practices, and organizational standards among countries, Germany was selected for a dedicated subcohort analysis due to its substantial dataset contribution (10.3%, the participating centers can be found in the Supporting Information S1) and distinct national features—such as the absence of a national pediatric anesthesia training program and registry, along with considerable local practice variability. This focused evaluation aimed to benchmark German data against the broader European cohort, identify strengths and weaknesses, and support targeted quality improvement initiatives and guideline development tailored to the German healthcare setting, ultimately optimizing anesthesia care for neonates.

### 1.2 Objectives

The ESAIC_CTN_NECTARINE study reported critical events requiring intervention in 35.2% of overall cases, with significant international variation in anesthesia practices. The German cohort was analyzed following approval from the ESAIC_CTN_NECTARINE Trial Steering Committee.

The primary objective of this analysis was to identify perioperative critical events that triggered medical interventions and explore their relationship to morbidity and mortality. Secondary endpoints included patient characteristics, procedures, and perioperative management (specifically anesthesia practice in Germany), identifying risk factors associated with critical events, and comparing the results with those of the broader European ESAIC_CTN_NECTARINE study, taking into account the considerable variability in pediatric anesthesia standards across Europe [9].

## 2 Methods

### 2.1 Study Design/Setting/Participants

ESAIC_CTN_NECTARINE (ClinicalTrials.gov NCT02350348) was a multi‐center observational study conducted over a 3‐month period across 31 European countries. Ethics approval for this study (Ethical Committee No. 374REG2015) was provided by the Ethical Committee of the University of Genoa, Italy (Chairperson Prof. Francesco De Stefano) on 13 October 2015. Recruitment took place from March 1, 2016, to January 31, 2017, and data were recorded using standardized case report forms (CRFs). The study included all children up to 60 weeks' postmenstrual age who underwent a surgical or diagnostic procedure requiring anesthesia or sedation.

Preoperative patient characteristics, intraoperative and postoperative anesthesiological management, as well as the occurrence of critical events and triggers for intervention, were recorded. Follow‐up assessments of morbidity and mortality were conducted at 30 and 90 days. The reporting of this study follows the STROBE (Strengthening the Reporting of Observational Studies in Epidemiology) guidelines.

The data were pseudonymized at the respective center site, anonymized, and electronically transmitted to the ESAIC_CTN_NECTARINE research team for analysis (OpenClinica, Boston, MA, USA).

### 2.2 Variables/Data Sources/Measurement

The primary objective of the Europe‐wide evaluation was to record critical events and identify the thresholds of physiological parameters that triggered intervention by the anesthesiologist.

The following parameters were defined:

- SpO_2_, PaO_2_, or both (intervention to improve oxygenation)
- End‐tidal carbon dioxide (ETCO_2_), arterial/venous blood CO_2_ (intervention to improve alveolar ventilation), or both
- Systolic or mean arterial blood pressure.
- Heart rate, ECG rhythm disturbances, or both resulting in cardiovascular instability
- Absolute values or relative changes in cerebral oxygenation when near‐infrared spectroscopy (NIRS) was part of clinical monitoring
- Blood glucose, plasma sodium (Na^+^), or both
- Hemoglobin values (requirement for packed red cell transfusion)
- Core body temperature values (correction for hypo/hyperthermia)

Additionally, data on patient characteristics, surgical or diagnostic procedures, perioperative anesthesiological management, and postoperative complications were analyzed.

The parameters listed in Tables S2 and S3/Figures 5 and 6 were selected for risk factor analysis in this study; they also correspond to those commonly found in the literature [10, 11, 12].

### 2.3 Study Size

**FIGURE 1:**
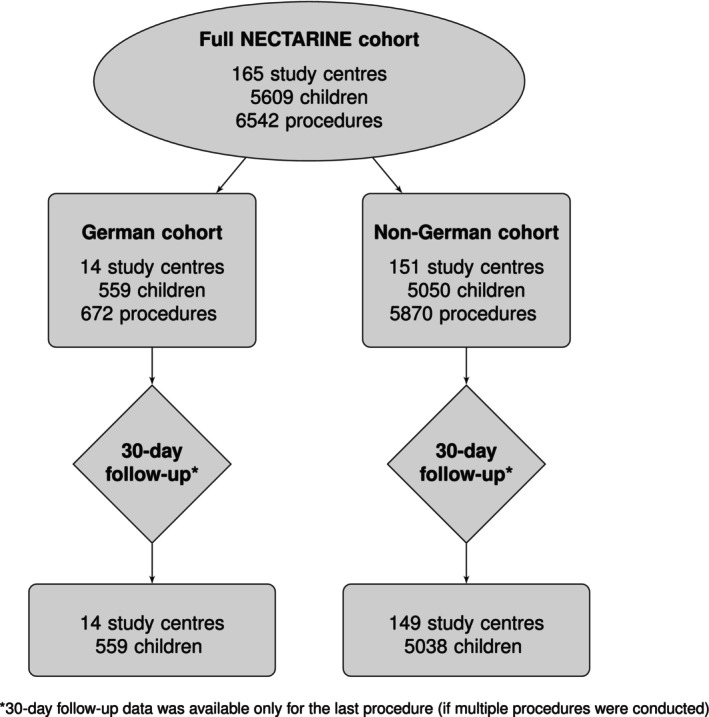
Summary of the number of study centers, patients, and procedures.

The ESAIC_CTN_NECTARINE study included a total of 6542 procedures involving 5609 children. In the German cohort, 672 procedures were performed in 559 children, while the non‐German cohort accounted for 5870 procedures involving 5050 children. Within the NECTARINE cohort, 651 children (11.6%) underwent more than one procedure during the 3‐month inclusion period (79 (14.1%) children in the German cohort and 572 (11.3%) in the non‐German cohort). For cases involving multiple procedures, 30‐day follow‐up data is available only for the final procedure. For further details, see Figure 1.

### 2.4 Statistical Methods

Statistical analyses were performed using the R language and environment for statistical computing (version 4.3.2). Descriptive statistics for continuous variables are presented as mean and standard deviation (mean ± SD) for normally distributed data, and median with interquartile range (median (IQR)) for skewed data. Categorical variables are presented as absolute numbers and valid frequencies. To compare the German and non‐German cohorts, an independent *t*‐test was applied for normally distributed continuous variables, while the Mann–Whitney *U* test was used for non‐normally distributed data. Categorical variables and relative frequencies were compared using the Chi‐squared (*χ* ^2^) test. Differences in percentages are presented with 95% confidence intervals calculated using the Newcombe–Wilson method. Analyses were performed either at the child or procedure level, depending on the variable of interest.

The impact of risk factors on the occurrence of critical events in the German sub cohort was analyzed using univariable and multivariable mixed‐effects logistic regression. Risk factors were treated as fixed effects, with a random intercept included to account for variability across study centers. For this analysis, only the last procedure for each patient was considered, adjusting for the number of surgeries. Odds ratios (ORs) with corresponding 95% CIs are reported for the fixed effects. Similarly, the impact of risk factors on morbidity and mortality was evaluated. Due to the small number of 30‐day mortality events, only the association between critical events and mortality was analyzed. The 30‐day mortality incidence was calculated as percentages, with 95% CIs derived using normal approximation. The significance level for all tests was set at 5%. Given the exploratory nature of the study, no adjustments were made for multiple testing.

### 2.5 Manuscript Preparation

Portions of the manuscript were refined for clarity using ChatGPT; no scientific content, data, or analyses were created or altered.

## 3 Results

### 3.1 Participants and Procedures

The baseline characteristics of the German cohort closely mirror those of the non‐German and overall NECTARINE cohorts, with similar metrics across all groups in terms of age, birth weight, sex, ASA status ≥ II, and pre‐existing conditions (see Tables 1 and 2). Notably, the percentage of cesarean sections is higher in the German cohort (German: 269/523 (51.4%), non‐German: 1984/4602 (43.1%); difference: 8.3 percentage points, 95% CI 2.6–14.0). Additionally, the German cohort has a higher proportion of elective procedures (German: 458/634 (68.2%), non‐German: 2946/5397 (50.2%); difference: 18.0 percentage points, 95% CI 13.1–22.6). For the majority of children in both cohorts, it represented the first surgical procedure associated with their episode of inpatient care (German: 480/559 (85.9%), non‐German: 4478/5050 (88.7%)).

**TABLE 1:** Baseline characteristics on children level.

	German (*N* = 559)	Non‐German (*N* = 5050)	NECTARINE full cohort (*N* = 5609)
Gestational age at birth (weeks)			
Median (IQR)	38.0 (33.0, 39.0)	38.0 (35.0, 39.0)	38.0 (35.0, 39.0)
*n*	559	5050	5609
Birth weight (g)			
Mean ± SD	2620.36 ± 1031.28	2742.50 ± 977.62	2730.38 ± 983.66
n	551	4999	5550
APGAR score at 5 min			
Median (IQR)	9.0 (8.0, 10.0)	9.0 (8.0, 10.0)	9.0 (8.0, 10.0)
*n*	397	3321	3718
Sex			
Male	379 (67.8%)	3291 (65.2%)	3670 (65.4%)
*n*	559	5050	5609
Congenital anomalies			
Yes	216 (38.6%)	2240 (44.4%)	2456 (43.8%)
*n*	559	5050	5609
Delivery			
Vaginal	254 (48.6%)	2618 (56.9%)	2872 (56.0%)
Cesarean section	269 (51.4%)	1984 (43.1%)	2253 (44.0%)
*n*	523	4602	5125
Surgery number			
1	480 (85.9%)	4478 (88.7%)	4958 (88.4%)
2	61 (10.9%)	427 (8.5%)	488 (8.7%)
3	9 (1.6%)	90 (1.8%)	99 (1.8%)
> 3	9 (1.6%)	55 (1.0%)	64 (1.1%)
*n*	559	5050	5609

Abbreviations: APGAR, appearance, pulse, grimace, activity and respiration; g, gram; IQR, interquartile range; min, minutes; *n*, number of valid observations; SD, standard deviation.

**TABLE 2:** Baseline characteristics on procedure level.

	German (*N* = 672)	Non‐German (*N* = 5870)	NECTARINE full cohort (*N* = 6542)
ASA status			
I	49 (7.3%)	708 (12.1%)	757 (11.6%)
II	369 (54.9%)	2779 (47.3%)	3148 (48.2%)
III	178 (26.5%)	1745 (29.8%)	1923 (29.4%)
IV	73 (10.9%)	597 (10.2%)	670 (10.3%)
V	3 (0.4%)	35 (0.6%)	38 (0.6%)
*n*	672	5864	6536
Transfer after			
PICU/NICU	302 (44.9%)	2565 (43.7%)	2867 (43.9%)
PACU discharge			
Intermediate care/High dependency unit	64 (9.5%)	523 (8.9%)	587 (9.0%)
Ward	302 (44.9%)	2684 (45.8%)	2986 (45.7%)
Other	4 (0.6%)	92 (1.6%)	96 (1.5%)
*n*	672	5864	6536
Urgency			
Elective	458 (68.2%)	2946 (50.2%)	3404 (52.0%)
Semi‐elective/Urgent	138 (20.5%)	2178 (37.1%)	2316 (35.4%)
Emergency	76 (11.3%)	745 (12.7%)	821 (12.6%)
*n*	672	5869	6541
Respiratory complications			
Yes	96 (14.3%)	1098 (18.7%)	1194 (18.3%)
*n*	671	5864	6535
Cardiovascular complications			
Yes	129 (19.3%)	1275 (21.8%)	1404 (21.5%)
*n*	669	5860	6529

Abbreviations: ASA, American Society of Anesthesiologists; *n*, number of valid observations; NICU, neonatal intensive care unit; PACU, post‐anesthesia care unit; PICU, pediatric intensive care unit.

Further details on baseline characteristics are provided in Tables 1 and 2.

In the German cohort, the proportion of surgical procedures was significantly higher than in the non‐German cohort (German: 577/672 (85.9%), non‐German: 4619/5869 (78.7%); difference: 7.2 percentage points, 95% CI 3.3–10.7; *χ* ^2^‐test: *p* < 0.001) with esophageal and gastrointestinal surgery being the most common in both cohorts. A detailed list of the surgical and non‐surgical procedures performed is provided in Table 3.

**TABLE 3:** Overview of procedure types (surgical and non‐surgical).

	German (*N* = 672)	Non‐German (*N* = 5870)	NECTARINE full cohort (*N* = 6542)
Surgical procedure	*n* = 577	*n* = 4615	*n* = 5192
Cardiac	44 (7.6%)	385 (8.3%)	429 (8.3%)
Dermatology	24 (4.2%)	117 (2.5%)	141 (2.7%)
ENT‐Plastic	27 (4.7%)	280 (6.1%)	307 (5.9%)
Genitourinary	41 (7.1%)	230 (5.0%)	271 (5.2%)
Minimally invasive	28 (4.9%)	429 (9.3%)	457 (8.8%)
Neuro	35 (6.1%)	260 (5.6%)	295 (5.7%)
Esophageal, gastro‐intestinal	333 (57.7%)	2614 (56.6%)	2947 (56.8%)
Ophthalmology	5 (0.9%)	113 (2.4%)	118 (2.3%)
Orthopedic	33 (5.7%)	157 (3.4%)	190 (3.7%)
Thoracic	7 (1.2%)	30 (0.7%)	37 (0.7%)
Non‐surgical procedure	*n* = 95	*n* = 1250	*n* = 1345
Angiography/embolization	1 (1.1%)	30 (2.4%)	31 (2.3%)
Biopsy	3 (3.2%)	44 (3.5%)	47 (3.5%)
Bronchoscopy	8 (8.4%)	139 (11.1%)	147 (10.9%)
Burns dressing	1 (1.1%)	2 (0.2%)	3 (0.2%)
Cardiac lab (percutaneous valvuloplasty, rashkind procedure)	11 (11.6%)	90 (7.2%)	101 (7.5%)
CT‐Scan	2 (2.1%)	37 (3.0%)	39 (2.9%)
Cystoscopy	9 (9.5%)	56 (4.5%)	65 (4.8%)
Gastroenterology	4 (4.2%)	93 (7.4%)	97 (7.2%)
Infiltration or punction	0 (0%)	34 (2.7%)	34 (2.5%)
MRI	33 (34.7%)	307 (24.6%)	340 (25.3%)
Ophthalmologic examination/Laser	1 (1.1%)	38 (3.0%)	39 (2.9%)
Pericardial or pleural drainage	0 (0%)	14 (1.1%)	14 (1.0%)
PICC line/Central venous/Broviac	6 (6.3%)	243 (19.4%)	249 (18.5%)
Other/non‐surgical	16 (16.8%)	123 (9.8%)	139 (10.3%)

Abbreviations: CT, computed tomography; ENT, ear, nose and throat; MRI, magnetic resonance imaging; *n*, number of valid observations; PICC, Peripherally Inserted Central Catheter.

### 3.2 Anesthesia Management

In the German cohort, 412 out of 672 procedures (61.3%) were performed with general anesthesia, 39 procedures (5.8%) used regional anesthesia alone, and a combination of both techniques was used in 221 procedures (32.9%). In the non‐German cohort, 3977 out of 5868 procedures (67.8%) were performed with general anesthesia, 177 procedures (3.0%) used regional anesthesia alone, and 1714 procedures (29.2%) combined general and regional anesthesia. The distribution of anesthesia techniques varied significantly between the two cohorts (*χ* ^2^‐test: *p* < 0.001).

For general anesthesia induction, multiple drugs were used in the majority of procedures, with 430 out of 632 (68.0%) in the German cohort and 3397 out of 5687 (59.7%) in the non‐German cohort. A single drug was used in 202 procedures (32.0%) in the German cohort and 2290 procedures (40.3%) in the non‐German cohort. The distribution of the number of drugs given for anesthesia induction differed significantly between the two cohorts (*χ* ^2^‐test: *p* < 0.001).

**FIGURE 2:**
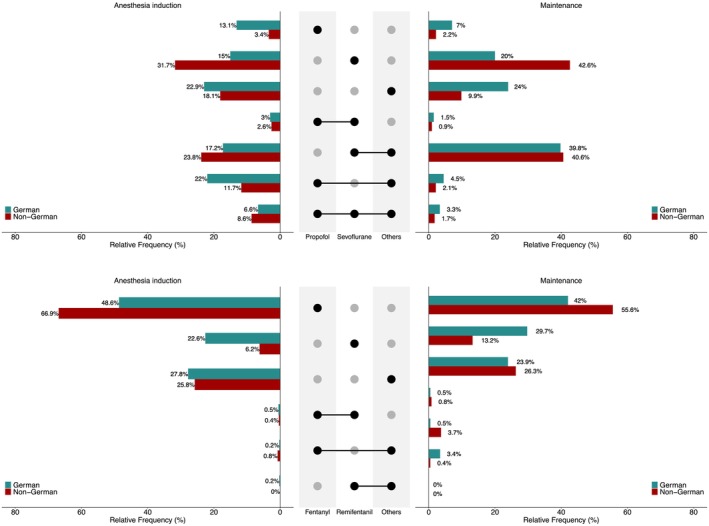
Distribution of drugs (upper panel) and opioids (lower panel) for anesthesia induction (left) and maintenance (right). The black dots in the middle represent the drugs (or opioids) and combinations, the bars represent the respective relative frequencies in the German and non‐German cohort. For example, Propofol was administered to 44.7% of the patients in the German cohort, which is calculated as 13.1% (Propofol only) + 3% (Propofol and Sevoflurane) + 22% (Propofol and others) + 6.6% (Propofol, Sevoflurane and others).

In the German cohort, Propofol (either alone or in combination with other drugs) was used significantly more frequently for anesthesia induction, in 44.7% (283/632) compared to 26.3% (1500/5687) in the non‐German cohort (difference: 18.4 percentage points, 95% CI 13.4–23.4; *χ* ^2^‐test: *p* < 0.001, see Figure 2). In contrast, Sevoflurane (either alone or in combination with other drugs) was administered significantly more frequently in the non‐German cohort, in 66.7% (3798/5698) of the procedures, compared to 41.9% (265/632) in the German cohort (difference: 24.7 percentage points, 95% CI 19.6–29.7; *χ* ^2^‐test: *p* < 0.001, see Figure 2). Other drugs used during anesthesia induction and maintenance, included thiopental, ketamine, atropine, and etomidate. Neuromuscular blocking agents were used for general anesthesia induction in 69.8% (442/633) in the German cohort compared to 56.4% (3210/5694) in the non‐German cohort (difference: 13.5 percentage points, 95% CI 8.5–18.2; *χ* ^2^‐test: *p* < 0.001).

Opioids were administered during anesthesia induction in 417 out of 633 procedures (65.9%) in the German cohort and in 3044 out of 6342 procedures (53.5%) in the non‐German cohort (difference: 17.9 percentage points, 95% CI 12.9–22.7; *χ* ^2^‐test: *p* < 0.001). Fentanyl, either alone or in combination with other drugs, was used less frequently in the German cohort compared to the non‐German cohort (German: 199/403 (49.3%), non‐German: 2064/3036 (68.0%); difference: 18.6 percentage points, 95% CI 12.1–25.1; *χ* ^2^‐test: *p* < 0.001). In contrast, Remifentanil was more commonly administered in the German cohort than in the non‐German cohort (German: 94/403 (23.3%), non‐German: 200/3036 (6.6%); difference: 16.7 percentage points, 95% CI 11.9–21.9; *χ* ^2^‐test: *p* < 0.001). The distribution of drugs and opioids used for anesthesia induction is shown in the left panel of Figure 2.

For anesthesia maintenance, the majority of procedures in both the German and non‐German cohorts involved multiple drugs, with a significantly higher percentage in the German cohort (German: 423/633 (70.4%), non‐German: 2796/5691 (51.2%); difference: 17.7 percentage points, 95% CI 12.6 to 22.5; *χ* ^2^‐test: *p* < 0.001). The use of Propofol and Sevoflurane for anesthesia maintenance was consistent with their use during anesthesia induction. Further details are provided in Figure 2. Opioids for maintenance were administered in 414 out of 633 procedures (65.4%) in the German cohort, compared to 2741 out of 6342 procedures (48.2%) in the non‐German cohort (difference: 22.2 percentage points, 95% CI 17.2–27.0; *χ* ^2^‐test: *p* < 0.001). The distribution of drugs and opioids used for anesthesia maintenance is shown in the right panel of Figure 2.

In both cohorts, oxygen + air was the most frequently used carrier gas, with a significantly higher usage in the German cohort (German: 592/632 (93.7%), non‐German: 4811/5690 (84.6%); difference: 9.1 percentage points, 95% CI 6.0–11.7; *χ* ^2^‐test: *p* < 0.001). Notably, oxygen + *N*_2_O was used as carrier gas in 9.9% of procedures (561/5690) in the non‐German cohort, compared to 1.3% (8/632) in the German cohort (difference: 8.6 percentage points, 95% CI 6.6–10.0; *χ* ^2^‐test: *p* < 0.001).

In Germany, vasopressors were administered significantly more frequently at the very start of anesthesia (German: 93/632 (14.7%), non‐German: 406/5678 (7.1%); difference: 7.6 percentage points, 95% CI 4.3–11.2; *χ* ^2^‐test: *p* < 0.001).

Among the 260 procedures in the German cohort involving either regional anesthesia alone or in combination with general anesthesia, caudal anesthesia was used in 211 procedures (81.2%, see Table 4). In contrast, caudal anesthesia was significantly less frequent in the non‐German cohort, with 802 out of 1891 procedures (42.4%) (difference: 38.7 percentage points, 95% CI 31.3–45.2; *χ* ^2^‐test: *p* < 0.001). Additionally, spinal anesthesia was used in 13 out of 260 procedures (5.0%) in the German cohort, compared to 196 out of 1891 procedures (10.4%) in the non‐German cohort (difference: 5.4 percentage points, 95% CI 0.7–8.9, *χ* ^2^‐test: *p* = 0.009). Catheters for continuous regional analgesia were significantly more frequently used in the non‐German cohort (German: 2/260 (0.8%), non‐German: 131/1891 (7.0%); difference: 6.2 percentage points, 95% CI 3.1–8.0; *χ* ^2^‐test: *p* < 0.001).

**TABLE 4:** Regional anesthesia technique.

	German (*N* = 260)	Non‐German (*N* = 1890)	NECTARINE full cohort (*N* = 2150)
Central nerve blocks			
Spinal	13 (5.0%)	196 (10.4%)	209 (9.7%)
Caudal	211 (81.2%)	802 (42.4%)	1013 (47.1%)
Lumbar epidural	2 (0.8%)	43 (2.3%)	45 (2.1%)
Thoracic epidural	0 (0%)	32 (1.7%)	32 (1.5%)
Penile block	0 (0%)	24 (1.3%)	24 (1.1%)
Thoracic, abdominal and inguinal blocks			
TAP	2 (0.8%)	71 (3.8%)	73 (3.4%)
Paraumbilical	0 (0%)	84 (4.4%)	84 (3.9%)
Ilio‐inguinal	4 (1.5%)	128 (6.8%)	132 (6.1%)
Peripheral blocks			
Upper limb	0 (0%)	6 (0.3%)	6 (0.3%)
Lower limb	0 (0%)	6 (0.3%)	6 (0.3%)
Intercostal	0 (0%)	10 (0.5%)	10 (0.5%)
Craniofacial	0 (0%)	54 (2.9%)	54 (2.5%)
Infiltration of the wound	25 (9.6%)	347 (18.4%)	372 (17.3%)
Other	3 (1.2%)	87 (4.6%)	90 (4.2%)

Abbreviation: TAP, transverse abdominis plane.

### 3.3 Critical Events

**FIGURE 3:**
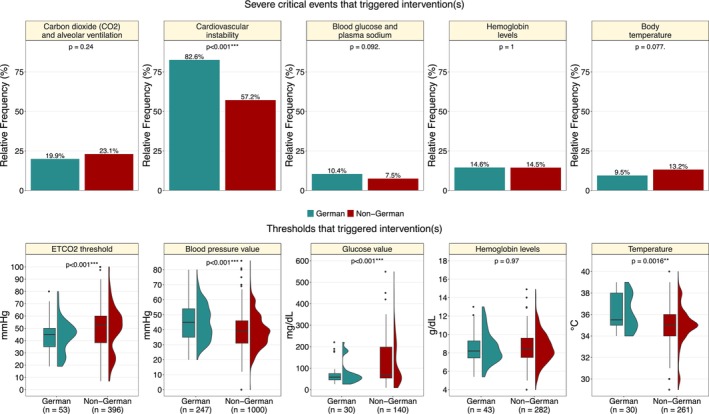
Relative frequencies (upper panel) and threshold values (lower panel) for interventions triggered by severe critical events. The *p*‐values in the upper panels refer to chi‐squared tests; the *p*‐values in the lower panels for the threshold values refer to two‐sided *t*‐tests for unpaired data.

The occurrence of at least one severe critical event requiring intervention was significantly more frequent in the German cohort, observed in 47.0% of the procedures (316/672), compared to 33.9% (1990/5869) in the non‐German cohort (difference: 13.1 percentage points, 95% CI 8.1–18.1; *χ* ^2^‐test: *p* < 0.001). Among procedures with critical events, the most common type was cardiovascular instability, occurring in 82.6% (261/316) of the cases in the German cohort and 57.2% (1138/1987) in the non‐German cohort (see Figure 3). Thus, cardiovascular instability occurred significantly more frequently in the German cohort with critical events (difference: 25.3 percentage points, 95% CI 18.6–31.3; *χ* ^2^‐test: *p* < 0.001). The corresponding mean arterial blood pressure (MAP, mmHg) threshold triggering intervention was significantly higher in the German cohort (German: 44.9 ± 11.5 vs. non‐German: 39.3 ± 11.4, *t*‐Test: *p* < 0.001), as shown in Figure 3. The temperature (°C) threshold triggering an intervention was also significantly higher in the German cohort (German: 36.0 ± 1.6 vs. non‐German: 35.0 ± 1.8, *t*‐Test: *p* = 0.0016). Conversely, the ETCO_2_ (mmHg) threshold was significantly lower in the German cohort (German: 43.3 ± 13.0 vs. non‐German: 50.5 ± 21.2, *t*‐Test: *p* < 0.001).

**FIGURE 4:**
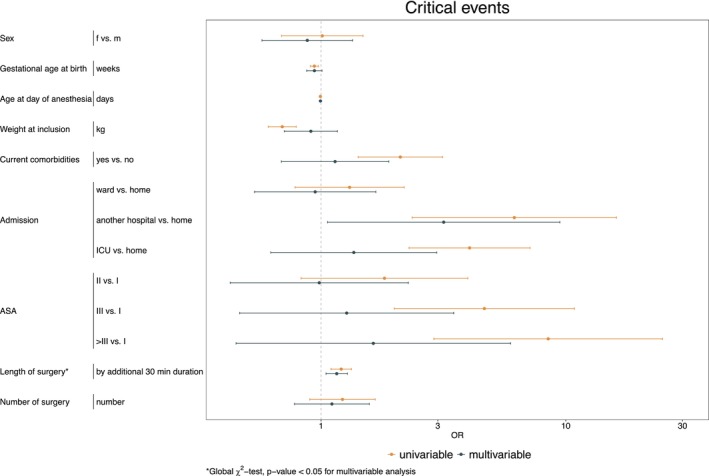
Results of univariable and multivariable mixed‐effects logistic regression for the occurrence of critical events requiring intervention.

Univariable mixed‐effects logistic regression on the German cohort (*n* = 559 children) identified gestational age at birth, age at day of anesthesia, weight, current comorbidities, admission, ASA, and the length of surgery as statistically significant factors influencing the occurrence of critical events. After adjusting for all considered risk factors, length of surgery was found to have a significant effect on the occurrence of severe critical events (see Figure 4 and Table S2).

### 3.4 30‐Day Morbidity and Mortality

**FIGURE 5:**
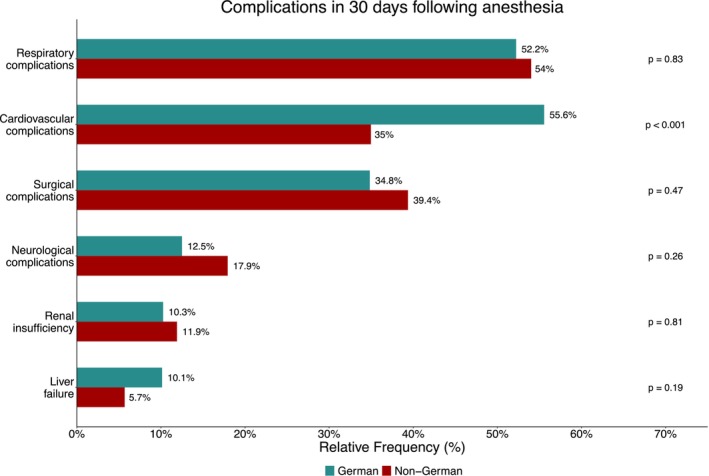
Relative frequencies of complications 30 days following anesthesia in the German and non‐German cohort. *p*‐values refer to a *χ* ^2^‐test.

Data from 545/559 (97.5%) children in the German cohort and 4678/5038 (92.9%) in the non‐German cohort were available for the 30‐day follow‐up. Morbidity was defined as the occurrence of one or more complications within 30 days following anesthesia. Complications occurred in 90 out of 493 (18.3%) children in the German cohort compared to 760 out of 4511 (16.8%) in the non‐German cohort (*χ* ^2^‐test: *p* = 0.4671). The most common complications in both cohorts were respiratory and cardiovascular complications (see Figure 5). Cardiovascular complications were significantly more frequent in the German cohort (German: 50/90 (55.6%), non‐German: 265/758 (35.0%); difference: 20.6 percentage points, 95% CI 6.8–33.7; *χ* ^2^‐test: *p* < 0.001). A detailed overview of 30‐day complications is provided in Figure 5.

**FIGURE 6:**
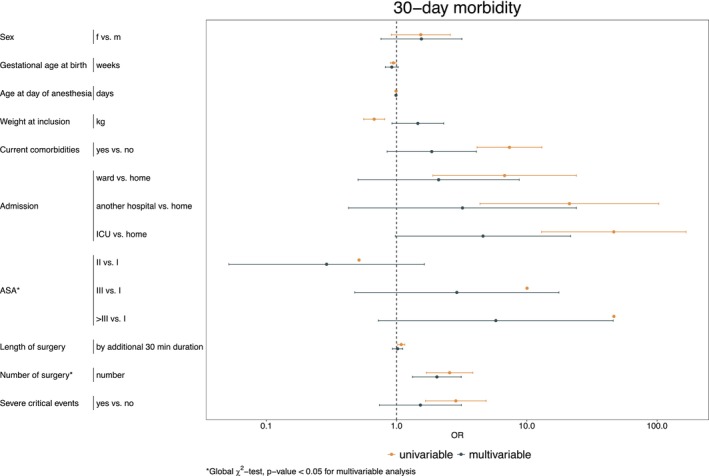
Results of univariable and multivariable mixed‐effects logistic regression for 30‐day morbidity. It should be noted that the confidence interval limits for the odds ratios of ASA status in the univariable analysis are extremely narrow, resulting in them not being visually distinguishable in this visualization.

A subgroup analysis of the German cohort (*n* = 493 children) with available 30‐day morbidity follow‐up data revealed a significantly higher risk of complications by day 30 if a critical event requiring intervention had occurred (unadjusted OR: 2.85 [95% CI 1.67–4.87]) (see Figure 6 and Table S3). Specifically, the likelihood of complications within 30 days was about three times higher following a critical event than in its absence. However, after adjusting for multiple risk factors (Figure 6 and Table S3), the occurrence of a critical event was not a statistically significant predictor on 30‐day morbidity. Multivariable mixed‐effects logistic regression identified ASA status and the surgery number as significant predictors of morbidity, while no statistically significant association was found for the remaining risk factors (see Figure 6 and Table S3).

The 30‐day incidence of mortality was 2.4% (95% CI 1.1%–3.7%) in the German cohort, compared to 2.0% (95% CI 1.6%–2.4%) in the non‐German cohort. A subgroup analysis of procedures performed in Germany (*n* = 658 procedures) showed that the risk of death within 30 days was 3.4 times higher following a critical event than in its absence (unadjusted OR: 3.4 (95% CI 1.41–9.49)). Between 30 and 90 days, the mortality rate was 0.2% (95% CI 0.0%–6.5%) in the German cohort and 0.8% (95% CI 0.5%–1.1%) in the non‐German cohort.

## 4 Discussion

Fourteen hospitals in Germany participated in the ESAIC_CTN_NECTARINE study, contributing 672 datasets (10.27% of the total analysis). No statistically significant differences were found between the German and non‐German cohorts in terms of age, gender, or ASA status.

In Germany, 29.6% of deliveries were by cesarean section, exceeding the World Health Organization's recommended medically necessary rate of no more than 10% [13]. Whether the higher cesarean rate reflects increased comorbidities and anesthetic risk remains elusive.

The distribution of interventions revealed that German centers performed a higher proportion of surgical procedures, which may have contributed to the increased incidence of critical events requiring intervention (47.0% in Germany vs. 33.9% in the non‐German cohort).

The choice of anesthetic drugs varied significantly. German anesthesiologists showed a clear preference for Propofol as a hypnotic, aligning with Sgrò et al.'s findings that it can be used for neonatal induction [14]. Total intravenous anesthesia offers benefits, such as reducing postoperative respiratory complications [15], but Propofol is frequently associated with hypotension, particularly in neonates. Simons et al. reported a 39% incidence of hypotension during Propofol‐induced intubation [16, 17], which may explain the more than twice as frequent use of vasopressors in the German cohort compared to the non‐German cohort (14.7% vs. 7.1%). The data from the German sub‐analysis indicate that the administration of Propofol, particularly in very small patients, should be approached with caution due to this adverse effect. Further discussion and evaluation within the relevant expert committees are warranted.

The original NECTARINE evaluation revealed variability in the anesthesiologists' intervention thresholds, often exceeding professional guidelines. In this sub‐analysis, the incidence of critical events requiring intervention was significantly higher in Germany (47.0%) compared to the non‐German cohort (35.2%), highlighting the need for root cause analysis. Cardiovascular instability was the most common complication (82.6% vs. 57.2%). Notably, German centers intervened at an average MAP of 44.9 ± 11.5 mmHg, aligning more closely with expert recommendations than the non‐German cohort (39.3 ± 11.4 mmHg) [18]. The higher incidence of cardiovascular complications in Germany, despite this adherence, may be partially attributed to the more frequent use of Propofol. Nonetheless, the application of a more stringent threshold for indicating intervention likely contributed to this finding. According to the Public Pediatric Assessment Report, Propofol use is neither contraindicated nor explicitly recommended for very young children (https://db.cbg‐meb.nl/pars/h110811.pdf). While its broader use in this sub‐analysis does not establish causality for increased cardiovascular complications, further studies are needed to refine recommendations for neonatal use.

Although data collection occurred before sustainability became a major focus in anesthesiology [19], Germany had already adopted a more restrictive approach to Sevoflurane and other volatile anesthetics, which significantly contribute to climate change [20]. Similarly, nitrous oxide, a potent greenhouse gas, is rarely used in Germany, whereas it remains relatively common in other countries (9.6%), underscoring the need for a global re‐evaluation of its use.

Remifentanil was the most commonly used opioid in Germany; it is favored for its controllability and safety in neonates due to its unique, organ‐ and age‐independent constant context‐sensitive half‐time [21].

The widespread use of regional anesthesia techniques in Germany reflects the integration of multimodal pain management concepts in clinical practice. Caudal anesthesia remains the most common and safest block, with ultrasound guidance proving valuable in cases of fetal anomalies [22]. While neuroaxial anesthesia is predominant in Germany, the use of peripheral nerve blocks is increasing, offering a safe option for anesthetizing children under sedation. In contrast, other European countries employ a broader range of regional anesthesia techniques, which could be beneficial for German patients in the future [23].

German centers followed stricter thresholds for temperature (≥ 36.0°C) and CO_2_ (≤ 43.3 mmHg), adhering to professional recommendations. Effective management of critical events requires prompt, individualized treatment and pre‐emptive consideration of risk factors such as ASA status and repeated surgeries.

The impact of critical events on postoperative outcomes highlights the importance of the attending anesthesiologist's experience, particularly in managing young and critically ill children. The NECTARINE study recorded only whether senior or junior anesthesiologists were present but did not capture years of expertise in pediatric anesthesia. In contrast, the *Anesthesia PRactice In Children Observational Trial* (APRICOT) study indicated that pediatric anesthesia expertise (measured in years) was generally higher across Europe than in Germany [1]. Nevertheless, German centers maintained high staffing standards, with senior anesthesiologists being present in 93.2% of procedures and nurse anesthesiologists in 99.6%.

Unlike Scandinavia, the UK, or France, Germany lacks formal subspecialty training in pediatric anesthesia. Pediatric anesthesia experts and the initiative Safetots.org recommend harmonizing training in pediatric anesthesia to improve outcomes [24]. To address this gap, several German hospitals started collaborating to standardize postgraduate pediatric anesthesia fellowship programs [25]. By integrating structured individual training and teaching in dedicated centers with institutional expertise and national scientific collaboration, these efforts aim to advance pediatric anesthesia in Germany.

There are still numerous scientific questions to address in order to improve outcomes in pediatric anesthesia. The large pan‐European observational studies, ESAIC_CTN_APRICOT and NECTARINE, were a crucial first step. The excellent European collaboration under the ESAIC umbrella continues with PARNet, representing the European Pediatric Anesthesia Research Network (https://esaic.org/research/research‐groups/parnet/). All pediatric anesthesia centers in Europe are encouraged and urged to participate in audits, studies and other scientific activities. Only in this way can the goal of providing optimal anesthetic care for children across the board be achieved.

## 5 Limitations

The study's observational design presents limitations, as it only analyzed interventions in response to critical events, without accounting for preventive measures. Additionally, treatment decisions were based solely on the attending anesthesiologist's judgment, relying on self‐reported data. Despite these limitations, the results provide valuable insights into anesthetic practices for neonates in Germany.

## 6 Conclusions

Caring for neonates poses significant challenges due to the frequency of perioperative critical events associated with increased morbidity and mortality. Rapid and effective intervention is essential to reduce morbidity and mortality in this vulnerable population. Harmonized pediatric anesthesia training and centralizing care in specialist centers would likely improve postoperative outcomes in this sensitive patient group.

## Funding

The authors have nothing to report.

## Conflicts of Interest

The authors declare no conflicts of interest.

## Supporting information


**Supporting Information S1:** NECTARINE centers and collaborator list.

**Table S2:** Results of univariable and multivariable mixed‐effects logistic regression for the occurrence of critical events requiring intervention.

**Table S3:** Results of univariable and multivariable mixed‐effects logistic regression for 30‐day morbidity.

## Data Availability

The data that support the findings of this study are available from the corresponding author upon reasonable request.
