# Supplementary material for: Epidemiology, Morbidity and Mortality Associated With Anesthesia in Early Life: A Subgroup Analysis of the German NEonate and Children audiT of Anesthesia pRactice IN Europe (NECTARINE) Cohort
Source: Paediatr Anaesth. 2026 Jan 16;36(4):440–52. doi: 10.1002/pan.70115 (PMC12972260; doi:10.1002/pan.70115)
Supplement: Supplementary file 1 — Supporting Information S1: NECTARINE centers and collaborator list. [file PAN-36-440-s003.docx]

**AUSTRIA (Maria Vittinghoff)**

1. Klinikum Klagenfurt, Christian **Breschan,** Rudolf **Likar***, Manuela **Platzer.**
2. Medical University Graz, Isole **Edelman**, Johanes **Eger**, Stefan **Heschl**, Brigitte **Messerer**, Maria **Vittinghof***.
3. University Innsbruck, Ruth **Kroess***, Martina **Stichlberger.**

**BELGIUM (Francis Veyckemans)**

1. Cliniques universitaires St Luc, David **Kahn***, Thierry **Pirotte**, Caroline **Pregardien,** Francis **Veyckemans.**
2. Grand Hôpital de Charleroi, France **Stevens*.**
3. Queen Paola Children's Hospital, Johan **Berghmans*.**
4. University Hospital Ghent, Annemie **Bauters**, Luc **De Baerdemaeker**, Stefan **De Hert***, Koen **Lapage**, Aliaksandra **Parashchanka**, Jurgen **Van Limmen**, Piet **Wyffels**.
5. University hospitals Leuven, Julie **Lauweryns*.**
6. Universitair Ziekenhuis Brussel (UZ Brussel), Nadia **Najafi*.**
7. Ziekenhuis Oost-Limburg, Joris **Vundelinckx*.**

**CROATIA (Sandra Kralik)**

1. Children's Hospital Zagreb, Diana **Butković**, Ivana **Kerovec Sorić**, Sandra **Kralik***, Ana **Markić**.
2. Rijeka University Hospital, Josip **Azman***.
3. University hospital Split, Josko **Markic**, Daniela **Pupacic***.

**CZECH REPUBLIC (Jiří Žurek)**

1. Faculty Hospital Ostrava**,** Michal **Frelich,** Petr **Reimer**, René **Urbanec*.**
2. Fakultni Nemocnice Motol, Petra **Cajková**, Vladimír **Mixa***.
3. University Hospital Brno, Yvona **Sedláčková**, Lenka **Knoppová,** Alena **Zlámalová (neé Květoňová)**, Martin **Vavřina***, Jiří **Žurek**.

**DENMARK (Tom Hansen)**

1. Odense University Hospital, Tom **Hansen***.
2. Rigshospitalet, Department of Anesthesia, Juliane Marie Center - University of Copenhagen, Arash **Afshari***, Anders **Bastholm Bille**, Marguerite **Ellekvist.**

**ESTONIA (Reet Kikas)**

1. Tallinn Children`s Hospital, Mari-Liis **Ilmoja***, Reet **Moor.**
2. Tartu University Hospital, Reet **Kikas***, Merle **Väli**.

**FINLAND (Tuula Manner)**

1. Helsinki University Central Hospital, Kariantti **Kallio**, Elisa **Reponen**, Pertti **Suominen,** Sami **Suvanto***, Raisa **Vähätalo**.
2. Kuopio University Hospital, Hannu **Kokki*,** Merja **Kokki.**
3. Tampere University Hospital, Jarkko **Harju**, Miia **Kokkonen**, Jenni **Vieri***.
4. Turku University Hospital, Tuula **Manner***.

**FRANCE (Christophe Dadure, Anne Lafargue)**

1. American Memorial Hospital CHU Reims, Catherine **Amory**, Hugues **Ludot***.
2. CHRU de Lille Hôpital Jeanne de Flandre, Dina **Bert**, Juliette **Godart**, Anne **Laffargue***.
3. CHU Amiens Picardie, Hervé **Dupont***, Benjamin **Urbina**.
4. CHU Bicetre, Catherine **Baujard***, Philippe **Roulleau**, Giuseppe **Staiti**.
5. CHU de Bordeaux, Maryline **Bordes**, Karine **Nouette Gaulain***, Yann **Hamonic**, François **Semjen**.
6. CHU de Nantes, Olivier **Jacqmarcq**, Caroline **Lejus-Bourdeau** Cécile **Magne***.
7. CHU Nancy, Léa **Petry**, Lilica **Ros**, Aurélien **Zang***.
8. CHU Toulouse, Mehdi **Bennis***, Bernard **Coustets***, Rose **Fesseau**.
9. Hopital Armand Trousseau, Isabelle **Constant**, Eliane **Khalil***, Nada **Sabourdin.**
10. Hopital couple enfant CHU de Grenoble, Noemie **Audren***, Thomas **Descarpentries**, Fanny **Fabre**, Aurélien **Legrand**.
11. Hôpital Universitaire Necker Enfants Malades, Emilie **Druot**, Gilles **Orliaguet***, Lucie **Sabau**, Lynn **Uhrig**.
12. Hôpitaux Pédiatriques de Nice CHU Lenval, François **de la Brière***, Karin **Jonckheer**, Jean-Paul **Mission**, Lucia **Scordo**.
13. Lapeyronie – CHU of Montpellier, Caroline **Couchepin**, Christophe **Dadure***, Pablo **De la Arena**, Laurent **Hertz**, Philippe **Pirat**, Chrystelle **Sola.**
14. Robert Debré University Hospital, Myriam **Bellon**, Souhayl **Dahmani***, Florence **Julien-Marsollier**, Daphne **Michelet**.
15. Teaching Hospital of Caen, Veronique **Depret-Donatien***, Anne **Lesage**.

**GERMANY (Karin Becke, Claudia Hoehne)**

1. Childrens Hospital Cologne, Jost **Kaufmann**, Michael **Laschat***, Frank **Wappler**.
2. Cnopf Childrens Hospital, Karin **Becke***, Lena **Brunner**, Karin **Oppenrieder**.
3. Klinik St. Hedwig, Gregor **Badelt**, Karin **Hochmuth**, Bernhard **Koller**, Anita **Reil**, Sebastian **Richter***.
4. Klinikum Kassel, Thomas **Fischer***.
5. Klinikum Oldenburg, med. Campus der Universität, Anja **Diers**, Clemens **Schorer**, Andreas **Weyland***.
6. Klinikum Stuttgart Olgahospital, Ruth **Cohausz**, Franz-Josef **Kretz***, Michaela **Löffler**, Markus **Wilbs**.
7. Universitaetsklinikum Leipzig, Claudia **Hoehne***, Johanna **Ulrici**.
8. University Hospital Münster, Christiane **Goeters***.
9. University Hospital Frankfurt/Main, Armin **Flinspach**, Matthias **Klages***, Simone **Lindau**, Leila **Messroghli**, Kai **Zacharowski**.
10. University Hospital Heidelberg, Christoph **Eisner**, Thomas **Mueller***, Daniel **Richter**, Melanie **Schäfer**, Markus **Weigand**, Sebastian **Weiterer**.
11. University Medical Centre Mannheim, Miriam **Ochsenreiter**, Michael **Schöler***, Tom **Terboven**
12. University Hospital of Cologne, Isabel **Eggemann**, Sascha **Haussmann**, Nicolas **Leister**, Christoph **Menzel**, Uwe **Trieschmann***, Sirin **Yücetepe**.
13. University of Wuerzburg, Susanna **Keilig**, Peter **Kranke***, Yvonne **Jelting**.
14. University of Bonn, Torsten **Baehner**, Richard **Ellerkmann***, Shahab **Ghamari**, Claudia **Neumann**, Martin **Söhle**.

**GREECE (Anna Malisiova)**

1. Democritus University of Thrace, Pelagia **Chloropoulou***.
2. "G. Gennimatas" General Hospital of Thessaloniki, Vagia **Ntritsou*,** Pinelopi **Papagiannopoulou**.
3. General Pediatric Hospital "Agia Sophia", Eleana **Garini**, Afroditi **Karafotia**, Panagoula **Mammi***.
4. Hippokrateio General Hospital, Thessaloniki, Evangelia **Bali**, Despoina **Iordanidou***
5. P & A Kyriakou Children Hospital, Anna **Malisiova***, Artemis **Polyzoi**, Adelais **Tsiotou.**

**HUNGARY (Andrea Székely)**

1. Gottsegen Hungarian Institute of Cardiology, Erzsebet **Sapi***, Edgar **Székely**.
2. Heim Pal National Pediatric Institute, Nandor **Kosik**, Veronika **Maráczi***, Janos **Schnur**.
3. Semmelweis University, Judit **Csillag**, János **Gál**, Gergely **Göbl**, Balázs **Hauser***, András **Petróczy**, Gyula **Tövisházi**.

**IRELAND (Brendan O’Hare)**

1. Children’s Health Ireland – Crumlin, Stuart **Blain**, Sarah **Gallagher**, Sinead **Harte**, Mandy **Jackson**, Emma **Meehan**, Zeenat **Nawoor**, Brendan **O’Hare***, Mark **Ross**.

**ITALY (Nicola Disma)**

1. A.O.R.N Santobono Pausilipon, Daniela **Lerro*.**
2. A.O.U. Policlinico- Vittorio Emanuele Catania, Marinella **Astuto***, Rita **Scalisi**.
3. AOU A. Meyer, Giulia **Frasacco**, Elena **Lenares***, Roberto **Leone**
4. Azienda Ospedaliera di Padova, Maurizia **Grazzini**, Carmelo **Minardi**, Nicola **Zadra***.
5. Azienda Ospedaliero Universitaria Ospedali Riuniti Di Foggia, Gilda **Cinnella**, Antonella **Cotoia**, Dario **Galante***.
6. Azienda Ospedaliero-universitaria Pisana, Brita **De Lorenzo**, Beate **Kuppers***.
7. Azienda ospedaliero-universitaria Policlinico S.Orsola Malpighi, Giulia **Bottazzi**, Fabio **Caramelli**, Maria Cristina **Mondardini***.
8. Bambino Gesù Children's Hospital, IRCCS, Emanuele **Rossetti**, Sergio **Picardo**, Alessandro **Vittori***.
9. Children Hospital Vittore Buzzi, Anna **Camporesi***.
10. Fondazione IRCCS Ca' Granda Ospedale Maggiore Policlinico, Edoardo **Calderini**, Laura Brigitta **Colantonio**, Simona Anna **Finamore***, Giuliana Anna **Porro**.
11. Istituto Giannina Gaslini, Rachele **Bonfiglio**, Nicola **Disma***, Svetlana **Kotzeva**, Leila **Mameli**, Camilla **Micalizzi**, Alessia **Montaguti**, Clelia **Zanaboni**.
12. Ospedale dei bambini Di Cristina, Anna **Guddo***.
13. Ospedale G. Salesi, Gerald Rogan **Neba***.
14. Ospedale Papa Giovanni XXIII, Moreno **Favarato**, Bruno Guido **Locatelli***, Micol **Maffioletti**, Valter **Sonzogni**.
15. Fondazione Policlinico A.Gemelli IRCCS- Università Cattolica del Sacro Cuore- Roma, Rossella **Garra,** Maria **Sammartino***, Fabio **Sbaraglia**.
16. Policlinico P. Giaccone. University of Palermo, Andrea **Cortegiani***, Alessandra **Moscarelli**.
17. kraliSection of Anesthesiology, Analgesia and Intensive Care, Elena **Attanasi**, Simonetta **Tesoro***.
18. Spedali Civili di Brescia, Cristina **Agapiti***, Francesca **Pinzoni**, Cesare **Vezzoli**.
19. University of Rome "Sapienza", Policlinico Umberto I, Rome, Italy, Federico **Bilotta***.

**LATVIA (Zane Straume)**

1. Children Clinical University Hospital, Arta **Barzdina**, Zane **Straume***, Anda **Zundane**.

**LITHUANIA (Laura Lukosiene)**

1. Lithuanian University of Health Sciences Medical Academy, Laura **Lukosiene***, Irena **Maraulaite**, Ilona **Razlevice**.

**LUXEMBOURG (Bernd Schmitz)**

1. Centre Hospitalier de Luxembourg, Bernd **Schmitz***.

**MALTA (Francis Borg)**

1. Mater Dei Hospital, Stephanie **Mifsud***.

**NETHERLANDS (Jurgen de Graaf)**

1. Amsterdam University Medical Center, University of Amsterdam, Carolin **Aehling**, Celia **Allison**, Rients **De Boer**, Dina **Emal**, Markus **Stevens***.
2. Erasmus MC- Sophia Children's Hospital, Marielle **Buitenhuis**, Inge **De Liefde**, Andreas **Machotta**, Gail **Scoones**, Lonneke **Staals***, Jeremy **Tomas**, Anouk **Van der Knijff-van Dortmont**.
3. Haga Teaching Hospital, Marianne **Veldhuizen***.
4. Leiden University Medical Center, David **Alders***.
5. MUMC, Wolfgang **Buhre**, Eva **Schafrat**, Jan **Schreiber**, Petronella Mari **Vermeulen***.
6. Radboudumc, Mark **Hendriks***, Sandra **Lako**, Marieke **Voet-Lindner**, Barbe **Pieters**, Gert-Jan **Scheffer**, Luc **Tielens**.
7. Universitair Medisch Centrum Groningen, Anthony R. **Absalom**, Margot **Bergsma**, Joke **De Ruiter**, Sascha **Meier**, Martin **Volkers***, Tjerk **Zweers**.
8. Amsterdam University Medical Center, VU University, Anne M. **Beukers**, Christa **Boer***, Jurgen **Dertinger**.
9. University Medical Center Utrecht, Sandra **Numan**, Bas **Van Zaane***.

**NORWAY (Wenche B Boerke)**

1. Oslo University Hospital, Rikshospitalet, Wenche B **Boerke***, Nil **Ekiz**, Kristoffer **Stensrud**.
2. Oslo University Hospital-Ullevål, Inger Marie **Drage***.
3. St. Olavs Hospital, Trondheim University Hospital, Erik Ramon **Isern*.**

**POLAND (Marzena Zielinska)**

1. Poznan University of Medical Sciences, Department of Pediatric Anesthesiology and Intensive Care, Alicja **Bartkowska-Sniatkowska***, Malgorzata **Grzeskowiak**, Magdalena **Juzwa-Sobieraj**, Jowita **Rosada-Kurasińska.**
2. Public Paediatric Teaching Hospital of Medical University of Warsaw, Artur **Baranowski**, Karina **Jakubowska**, Dorota **Lewandowska**, Magdalena **Mierzewska-Schmidt***, Piotr **Sawicki**, Magdalena **Urban-Lechowicz.**
3. Wroclaw Medical University, Pomianek **Przemyslaw**, Marzena **Zielinska***.

**PORTUGAL (Maria Domingas Patuleia)**

1. Centro Hospitalar do Porto**,** Teresa **Leal**, Maria **Soares**, Pedro **Pina***, Sílvia **Pinho**.
2. Centro Hospitalar Lisboa Norte - Hospital de Santa Maria, Maria Domingas **Patuleia***.
3. Centro Hospitalar de Lisboa Central - Hospital D. Estefânia, Catarina **Cruz** **Esteves***.
4. Hospital de Braga, Helena **Salgado***, Maria João **Santos**.

**ROMANIA (Radu Tabacaru)**

1. CHILDREN HOSPITAL 'LOUIS TURCANU", Rodica **Badeti***.
2. Emergency Clinical Hospital of Constanta, Iulia **Cindea***.
3. Emergency Hospital for Children Cluj Napoca, Loredana **Oana***.
4. Spitalul de copii "Maria Curie", Adriana **Gurita**, Luminita **Ilie**, Gabriel **Mocioiu**, Radu **Tabacaru***, Irina **Trante**.
5. St. Maria" Children' s Hospital-Iasi", Valentin **Munteanu***.
6. Tirgu Mures Clinical Emergency Hospital, Mihai **Morariu***, Emese **Nyíri**.

**SERBIA (Dusica Simic)**

1. Clinic for Anesthesiology and Intensive Therapy, Clinical Centre Nis, Medical Faculty University of Nis, Ivana **Budic***, Vesna **Marjanovic**.
2. Children and Youth Health Care Institute of Vojvodina, Biljana **Drašković**, Marina **Pandurov**.
3. Mother and Child Healthcare Institute of Serbia, Jordanka **Ilic**, Ana **Mandras**, Zdenka **Rados**, Nikola **Stankovic***, Maja **Suica**, Sladjana **Vasiljevic**.
4. University Children s Hospital, Belgrade, Mirjana **Knezevic**, Irina **Milojevic***, Ivana **Petrov**, Selena **Puric Racic**, Dusica **Simic**, Irena **Simic**, Marija **Stevic**, Irena **Vulicevic**.

**SLOVAKIA (Miloslav Hanula)**

1. University Children hospital, Banská Bystrica, Barbora **Cabanová***, Miloslav **Hanula**.

**SLOVENIA (Jelena Berger)**

1. University Clinical Centre Ljubljana, Jelena **Berger***, Darja **Janjatovic**, Špela **Pirtovšek** **Štupnik**.

**SPAIN (Ignacio Gálvez Escalera)**

1. Hospital 12 de Octubre, Dolores **Méndez***, Gema **Pino**, Paloma **Rubio.**
2. Hospital Universitari Parc Taulí Sabadell, Alberto **Izquierdo**, Silvia **López***.
3. Hospital Universitario Donostia, Cristina **González Serrano***.
4. Hospital General Universitario Gregorio Marañón, Jesús **Cebrián***, Ana **Peleteiro**.
5. Hospital Infantil Universitario Niño Jesús, Pilar **Del Rey de Diego**, Ernesto **Martínez García***, Carolina **Tormo de las Heras**, Pablo **Troncoso Montero**.
6. Hospital Sant Joan De Déu, Celia **Arbona**, David **Artés**, Alicia **Chamizo**, Silvia **Serrano**, Montserrat **Suarez Comas***.
7. Hospital Universitari i Politècnic la Fe, Francisco **Escribá***.
8. Hospital Universitari Son Espases, Cristina **Auli***.
9. Hospital Universitario Marqués de Valdecilla, Osvaldo **Pérez** **Pardo**, Natalia **Sierra Biddle**, Ceferina **Suárez Castaño***, María Isabel **Villalobos Rico.**
10. Vall d'Hebron Barcelona Hospital Campus, Susana **Manrique Muñoz,** Irene **García Martínez***, Nuria **Montferrer Estruch,** Elena **Vilardell Ortíz**.
11. Hospital Clínico Universitario de Valladolid, Rodrigo **Poves-Álvarez.**

**SWEDEN (Albert Castellheim)**

1. Astrid Lindgren Children's Hospital, Ivan **Kohn***, Ulf **Lindestam**, Jarl **Reinhard**.
2. Queen Silvia Children´s Hospital, Sahlgrenska University Hospital, Albert **Castellheim***, Kerstin **Sandström**.
3. Uppsala University Hospital, Sporre **Bengt**, Rainer **Dörenberg**, Peter **Frykholm***, Maria **Garcia**, Ann **Kvarnström**, Emma **Pontén**.

**SWITZERLAND (Walid Habre)**

1. Bern University Hospital, Thomas **Bruelisauer**, Gabor **Erdoes**, Heiko **Kaiser**, Mathias **Marchon**, Thomas **Riva**, Stefan **Seiler***.
2. Centre Hospitalier Universitaire Vaudois, Yann **Bögli**, Mirko **Dolci***, Carine **Marcucci**.
3. Geneva Children's Hospital, Walid **Habre***, Isabelle **Pichon**, Laszlo **Vutskits**.
4. Kantonsspital Luzern, Mattias **Casutt**, Martin **Hölzle**, Thomas **Hurni**, Martin **Jöhr**, Anna-Ursina **Malär**, Jacqueline **Mauch***.
5. University Children's Hospital Basel, Thomas **Erb***, Karin **Oeinck**.

**TURKEY (Dilek Özcengiz - Zehra** **Hatipoğlu)**

1. Ankara Children's Health and disease Haematology Oncology Training And Research Hospital, Mine **Akin**, Gulsen **Keskin**, Yesim **Senayli***.
2. Cerrahpasa Medical School, Guner **Kaya**, Pinar **Kendigelen**, Ayse Çiğdem **Tutuncu***
3. Çukurova University, Zehra **Hatipoğlu**, Dilek **Özcengiz***.
4. Dokuz Eylül University, Hale Aksu **Erdost**, Elvan **Öçmen**, Çimen **Olguner***.
5. Marmara University, Hilmi **Ayanoglu**, Pelin Corman **Dincer**, Tumay **Umuroglu***.
6. Mersin University - Anaesthesiology, Mustafa **Azizoglu,** Handan **Birbiçer***, Nurcan **Doruk**, Aslı **Sagun**.
7. Ondokuz Mayis University, Sibel **Baris***.

**UKRAINE (Dmytro** **Dmytriiev)**

1. Vinnitsa national medical university, Dmytro **Dmytriiev***.

**UNITED KINGDOM (Thomas Engelhardt, Suellen Walker)**

1. Alderhey Childrens Hospital, Sridevi **Kuchi***, Nuria **Masip**.
2. Chelsea & Westminster Hospital, Peter **Brooks***, Alison **Hare**.
3. Great Ormond St Hospital, Nargis **Ahmad**, Michelle **Casey**, Sam **De Silva**, Nadine **Dobby**, Prakash **Krishnan**, L. Amaki **Sogbodjor**, Ellie **Walker**, Suellen **Walker***.
4. Guy's and St Thomas's NHS Trust, Stephanie **King**, Katy **Nicholson***, Michelle **Quinney**, Paul **Stevens**.
5. Kings College Hospital, Andrew **Blevin**, Mariangela **Giombini**, Chulananda **Goonasekera***.
6. Leeds Children's Hospital, Sadia **Adil**, Stephanie **Bew**, Carol **Bodlani**, Dan **Gilpin**, Stephanie **Jinks**, Nalini **Malarkkan**, Alice **Miskovic**, Rebecca **Pad**, Juliet **Wolfe Barry***.
7. Nottingham University Hospital, Joy **Abbott**, James **Armstrong***, Natalie **Cooper**, Lindsay **Crate**, John **Emery**, Kathryn **James**, Hannah **King**, Paul **Martin**.
8. Royal Aberdeen Children's Hospital, Stefano **Scalia Catenacci***.
9. Royal Alexandra Childrens' Hospital, Rob **Bomont**, Paul **Smith***.
10. Royal Brompton Hospital, Sara **Mele***, Alessandra **Verzelloni**.
11. Royal Devon and Exeter NHS Foundation Trust, Philippa **Dix***.
12. Royal Hospital for Sick Children Glasgow, Graham **Bell***, Elena **Gordeva**, Lesley **McKee**, Esther **Ngan**, Jutta **Scheffczik**, Li-En **Tan**, Mark **Worrall**.
13. Royal London Hospital, Carmel **Cassar***, Kevin **Goddard**.
14. Royal Manchester Children's Hospital, Victoria **Barlow**, Vimmi **Oshan***, Khairi **Shah**.
15. Royal Victoria Infirmary, Sarah **Bell**, Lisa **Daniels**, Monica **Gandhi***, David **Pachter**, Chris **Perry**, Andrew **Robertson**, Carmen **Scott**, Lynne **Waring**.
16. St Georges University Hospitals NHS Foundation Trust, David **Barnes**, Sophie **Childs**, Joanne **Norman**, Robin **Sunderland***.

Management Team

- European Society of Anaesthesiology, Brussels: **Dowell** Julia, **Feijten** Prisca, **Harlet** Pierre, **Herbineaux** Sarah, **Leva** Brigitte, **Plichon** Benoît, **Virág** Katalin.
