# Supplementary material for: Epidemiology, Morbidity and Mortality Associated With Anesthesia in Early Life: A Subgroup Analysis of the German NEonate and Children audiT of Anesthesia pRactice IN Europe (NECTARINE) Cohort
Source: Paediatr Anaesth. 2026 Jan 16;36(4):440–52. doi: 10.1002/pan.70115 (PMC12972260; doi:10.1002/pan.70115)
Supplement: Supplementary file 2 — Table S2: Results of univariable and multivariable mixed‐effects logistic regression for the occurrence of critical events requiring intervention. [file PAN-36-440-s002.pdf]

Table S2: Results of univariable and multivariable mixed-effects logistic regression for the occurrence of critical events requiring intervention.

**Risk factor**		**Univariable**			**Multivariable**		
		**N**		**OR (95% CI)**	**N**		**OR (95% CI)**
Sex female	n (%)	559	180 (32.2%)	1.01 (0.69, 1.48)	553	177 (32.0%)	0.88 (0.57, 1.35)
Gestational age at birth (weeks)	Median (IQR)	559	38.00 (33.00, 39.00)	0.94 (0.90, 0.98)	553	38.00 (33.00, 39.00)	0.94 (0.87, 1.01)
Age at day of anesthesia (days)	Median (IQR)	559	59.00 (27.00, 95.50)	0.99 (0.99, 1.00)	553	58.00 (27.00, 94.00)	0.99 (0.99, 1.00)
Weight at inclusion (kg)	Mean ± SD	557	4.10 ± 1.55	0.69 (0.61, 0.79)	553	4.10 ± 1.55	0.91 (0.71, 1.17)
Current comorbidities present	n (%)	555	187 (33.7%)	2.11 (1.42, 3.14)	553	185 (33.5%)	1.14 (0.69, 1.89)
Admission							
Home			150 (26.8%)	-		148 (26.8%)	-
Ward	n (%)	559	249 (44.5%)	1.31 (0.78, 2.18)	553	246 (44.5%)	0.95 (0.53, 1.67)
Another Hospital			29 (5.2%)	6.16 (2.36, 16.11)		28 (5.1%)	3.17 (1.06, 9.46)
ICU			121 (23.4%)	4.04 (2.29, 7.15)		131 (23.7%)	1.36 (0.62, 2.97)
ASA	n (%)						
I			46 (8.2%)	-		45 (8.1%)	-
II		559	329 (58.9%)	1.82 (0.83, 3.98)	553	324 (58.6%)	0.98 (0.43, 2.27)
III			142 (25.4%)	4.65 (1.99, 10.85)		142 (25.7%)	1.27 (0.46, 3.48)
>III			42 (7.5%)	8.48 (2.89, 24.86)		42 (7.6%)	1.64 (0.45, 5.95)
Length of surgery (minutes)*	Median (IQR)	559	47.00 (30.00, 88.50)	1.21 (1.10, 1.33)[§]	553	47.00 (30.00, 86.00)	1.16 (1.05, 1.28)[§]
Surgery number	Median (IQR)	559	1.00 (1.00, 1.00)	1.22 (0.90, 1.66)	553	1.00 (1.00, 1.00)	1.11 (0.78, 1.58)

*Global X^2-test, p-value < 0.05 for multivariable analysis; [§]OR (95% CI) for length of surgery is presented for an increase of 30 minutes. Abbreviations: ASA = American Society of Anesthesiologists, CI = Confidence Interval, ICU = Intensive Care Unit, IQR = Interquartile Range, OR = Odds Ratio, SD = Standard Deviation, kg = Kilogram
