# Supplementary material for: Epidemiology, Morbidity and Mortality Associated With Anesthesia in Early Life: A Subgroup Analysis of the German NEonate and Children audiT of Anesthesia pRactice IN Europe (NECTARINE) Cohort
Source: Paediatr Anaesth. 2026 Jan 16;36(4):440–52. doi: 10.1002/pan.70115 (PMC12972260; doi:10.1002/pan.70115)
Supplement: Supplementary file 3 — Table S3: Results of univariable and multivariable mixed‐effects logistic regression for 30‐day morbidity. [file PAN-36-440-s001.pdf]

Table S3: Results of univariable and multivariable mixed-effects logistic regression for 30-day morbidity.

| Risk factor | | Univariable | | | Multivariable | | |
|---|---|---|---|---|---|---|---|
| | | N | | OR (95% CI) | N | | OR (95% CI) |
| Sex female | n (%) | 493 | 126 (32.9%) | 1.53 (0.91, 2.58) | 487 | 159 (32.6%) | 1.55 (0.76, 3.17) |
| Gestational age at birth (weeks) | Median (IQR) | 493 | 38.00 (33.00, 39.00) | 0.95 (0.9, 0.99) | 487 | 38.00 (33.00, 39.00) | 0.92 (0.82, 1.03) |
| Age at day of anaesthesia (days) | Median (IQR) | 493 | 60.00 (27.00, 98.00) | 0.99 (0.99, 1) | 487 | 60.00 (27.00, 97.00) | 0.99 (0.98, 1.00) |
| Weight at inclusion (kg) | Mean ± SD | 491 | 4.09 ± 1.56 | 0.67 (0.56, 0.81) | 487 | 4.09 ± 1.56 | 1.46 (0.92, 2.30) |
| Current comorbidities present | n (%) | 489 | 165 (33.7%) | 7.36 (4.15, 13.04) | 487 | 163 (33.5%) | 1.86 (0.85, 4.10) |
| Admission | | | | | | | |
| Home | | | 116 (23.5%) | - | | 114 (23.4%) | - |
| Ward | n (%) | 493 | 238 (48.3%) | 6.75 (1.9, 24.01) | 487 | 235 (48.2%) | 2.10 (0.51, 8.74) |
| Another Hospital | | | 23 (4.7%) | 21.23 (4.37, 103.09) | | 22 (4.5%) | 3.21 (0.43, 24.0) |
| ICU | | | 116 (23.5%) | 46.5 (12.96, 166.82) | | 116 (23.8%) | 4.61 (0.98, 21.70) |
| ASA* | n (%) | | | | | | |
| I | | | 41 (8.3%) | - | | 40 (8.2%) | - |
| II | | 493 | 287 (58.2%) | 0.52 (0.51, 0.52) | 487 | 282 (57.9%) | 0.29 (0.05, 1.63) |
| III | | | 129 (26.2%) | 10.04 (10.00, 10.08) | | 129 (26.5%) | 2.90 (0.48, 17.6) |
| >III | | | 36 (7.3%) | 46.59 (46.4, 46.78) | | 36 (7.4%) | 5.78 (0.73, 46.00) |
| Length of surgery (minutes) | Median (IQR) | 493 | 47.00 (30.00, 90.00) | 1.09 (1.02, 1.16)§ | 487 | 47.00 (30.00, 89.50) | 1.02 (0.93, 1.11)§ |
| Surgery number* | Median (IQR) | 493 | 1.00 (1.00, 1.00) | 2.56 (1.7, 3.85) | 487 | 1.00 (1.00, 1.00) | 2.04 (1.33, 3.14) |
| Severe critical event | n (%) | 493 | 227 (46.0%) | 2.85 (1.67, 4.87) | 487 | 224 (46.0%) | 1.53 (0.74, 3.15) |

*Global $X^2$-test, p-value < 0.05 for multivariable analysis; §OR (95% CI) for length of surgery is presented for an increase of 30 minutes. Abbreviations: ASA = American Society of Anesthesiologists, CI = Confidence Interval, ICU = Intensive Care Unit, IQR = Interquartile Range, OR = Odds Ratio, SD = Standard Deviation, kg = Kilogram
